# A Comprehensive Assessment of the Marginal Abatement Costs of CO_2_ of Co-Optima Multi-Mode Vehicles

**DOI:** 10.1021/acs.energyfuels.4c03451

**Published:** 2024-12-19

**Authors:** Nicholas A. Carlson, Michael S. Talmadge, George G. Zaimes, Troy R. Hawkins, Yuan Jiang

**Affiliations:** † National Renewable Energy Laboratory, 15013 Denver West Parkway, Golden, Colorado 80401, United States; ‡ Argonne National Laboratory, 9700 Cass Avenue, Lemont, Illinois 60439, United States; § Pacific Northwest National Laboratory, 902 Battelle Boulevard, Richland, Washington 99354, United States

## Abstract

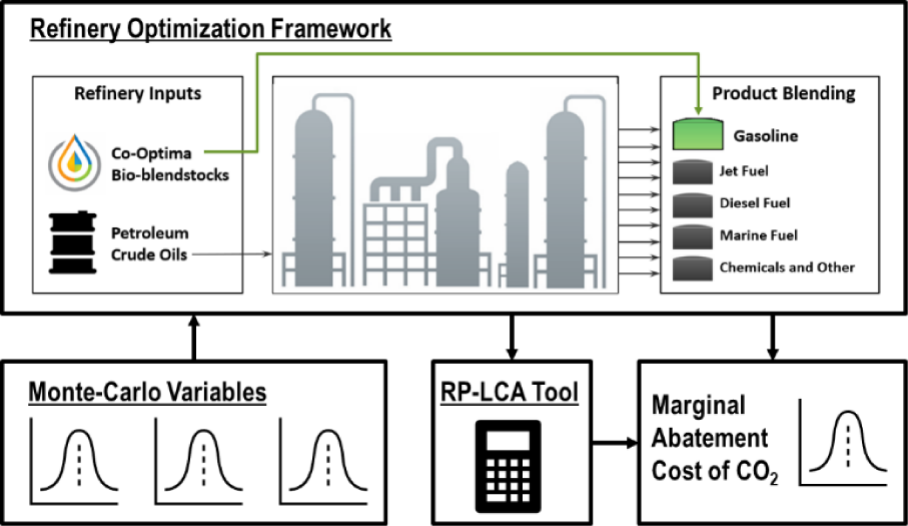

The Co-Optimization of Fuels and Engines (Co-Optima) is a research and development consortia funded by the U.S. Department of Energy, which has engaged partners from national laboratories, universities, and industry to conduct multidisciplinary research at the intersection of biofuels and combustion sciences. Since 2016, the Co-Optima team has examined high-quality bioblendstocks, and their properties, as design variables for increasing efficiency in modern engines while decarbonizing on-road light- and heavy-duty vehicles. The objective of this analysis is to combine and expand upon research into Co-Optima multi-mode bioblendstocks, which blend with petroleum gasoline to form high efficiency fuels for combustion in both spark ignition and advanced compression ignition engines. Consequently, the economic and environmental impacts of deploying 10 different multi-mode bioblendstocks derived from renewable and circular resources are quantified. Each bioblendstock is evaluated across several variables including (1) target blend levels of 10, 20, and 30 vol %, (2) years from 2030 to 2050, (3) crude oil benchmark prices, (4) vehicle lifetime miles, and (5) incremental vehicle costs. A Monte Carlo simulator is developed using a refinery optimization model and life-cycle analysis tool from prior Co-Optima research to sample marginal abatement costs of CO_2_, or cost of removing an additional unit of CO_2_, corresponding to each bioblendstock while considering input variable uncertainties. Results show that the combination of efficiency gains from advanced multi-mode fuel-engine technologies and the reoptimization of refinery operations results in several bioblendstocks demonstrating near-zero expected marginal abatement costs. Variable importances are also explored to highlight which aspects of the multi-mode technology are most influential in determining marginal abatement costs. Results suggest that Co-Optima multi-mode technology could provide economically viable decarbonization contributions to electrification-resistant light-duty vehicle sectors or near-term emission reductions, while Co-Optima fuels or alternatives decarbonize further to reach net-zero status.

## 1 Introduction

The Co-Optimization of Fuels and Engines (Co-Optima) initiative has sought to codesign fuels and engines to reduce emissions while achieving increased efficiencies with decarbonized fuels produced from renewable and circular sources. Since the initiative’s inception in 2016, the interest in, and volume of research on, alternative pathways toward decarbonizing transportation, such as vehicle electrification, has grown significantly. However, as with all decarbonization strategies, there are key barriers to electrifying light- and heavy-duty vehicles, including decarbonizing the grid, manufacturing batteries, and building charging networks at scale.^1^ These challenges naturally prompt questions about the role of decarbonized liquid fuels, like those developed in Co-Optima, in the transition toward a more sustainable transportation industry.

Several features of Co-Optima would likely make its deployment a seamless and near-term contribution to U.S. decarbonization goals. First, relatively minor engine modifications, mostly involving control strategy development, engine recalibration, and possible ignition system additions, would be required from existing manufacturers with incremental costs over traditional boosted spark ignition (BSI) vehicles estimated to be below $3000.^2,3^ Second, petroleum refineries could leverage their trillions in spent capital to integrate high-quality biofuels with minimal expense while potentially unconstraining operations, synchronizing with market trends, and unlocking new value streams.^4^ Third, utilizing existing transportation fuel infrastructure would greatly reduce capital investment requirements and would leave few barriers to consumer adoption.^5−7^

Prior research has produced experimental data and models to individually analyze each part of a hypothetical Co-Optima supply chain.^4,5,8,9^ As a continuation, this analysis seeks to combine the benefits identified in prior research associated with each supply chain element to comprehensively assess the value proposition of Co-Optima. Marginal abatement costs of CO_2_ (MAC), a common metric used in other decarbonization studies defined as the economic penalty incurred while preventing the emission of an addition unit of CO_2_ relative to a business-as-usual benchmark, are quantified for various scenarios. Calculating MACs normalizes costs by the degree of greenhouse gas (GHG) reduction for easy cost–benefit comparisons with other decarbonization pathways.

Although various Co-Optima fuels have been designed for light-, medium-, and heavy-duty vehicles, a subset of light-duty gasoline blendstocks, referred to as multi-mode (MM) gasolines for their ability to increase combustion efficiencies in both traditional spark ignition and advanced compression ignition engines, are placed into focus. Uncertainties still surround many variables that would influence Co-Optima’s success at the R&D stage. Consequently, a Monte Carlo framework is developed around the MAC calculation to ease the severity of assumptions while providing insights into the potential risks undertaken by producers. Results show the distributions of MACs that could be achieved by scaling the production and integration of each MM bioblendstock candidate. A machine learning model is trained to predict MAC based on major inputs across MM bioblendstocks to analyze variable importance and identify which technology improvements could be most impactful in further reducing MACs. Results suggest that Co-Optima MM technologies could provide near-term, economically viable decarbonization contributions to the light-duty vehicle sector while deeper decarbonization technologies, such as electrification with grid decarbonization, are adopted to ultimately reach net-zero emissions.

## 2 Methods

### 2.1 Marginal Abatement Cost Calculation

MACs were determined by calculating lifetime cost and emission differences associated with a consumer purchasing and operating an MM vehicle instead of a traditional boosted spark ignition (boosted-SI) light-duty vehicle. Co-Optima researchers developed a merit function to estimate Co-Optima engine efficiency gains over traditional boosted-SI engines (*EFF*_*Gain*_) as a function of fuel properties.^8^ Through experimentation, the research octane number (*RON*_*Blend*_) and sensitivity, defined as the difference between research and motor octane numbers (*S*_*Blend*_ = *RON*_*Blend*_*– MON*_*Blend*_), of the blended fuel were identified as the dominant fuel properties determining efficiency gains. The merit function is shown in simplified form in eq 1 where (*EFF*_*Bias*_) is a bias term accounting for terms unrelated to *RON*_*blend*_ or *S*_*Blend*_, which have a lesser impact on efficiency gains.^8^
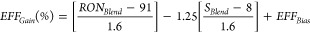
1

Consequently, the MM vehicle fuel efficiency (*FE*_*MM*_) could be predicted using eq 2 given a traditional boosted-SI vehicle’s fuel efficiency assumed to be 38 MPG in other Co-Optima research.^10^
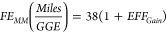
2

If the number of lifetime vehicle miles traveled (*LVMT*) by the MM and boosted-SI vehicle is assumed to be equal, lifetime fuel consumption (*LFC*) can be calculated as shown in eqs 3 and 4.
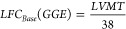
3
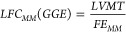
4

Next, given *LFCs*, the lifetime costs (*LTCs*) of operating each vehicle could be determined if the prices of each fuel were known. Given the price of a baseline gasoline (*P*_*Base*_), the minimum selling price the refinery would need to charge for MM gasoline (*MSP*_*MM*_) to maintain its baseline gross margin after integrating a bioblendstock was calculated using eq 5. In eq 5, *GM*_*Base*_ is the gross margin of the refinery while not producing MM gasoline, *GM*_*MM*_ is the gross-margin while producing MM gasoline, and *V*_*MM*_ is the volume of MM gasoline produced at price *P*_*Base*_. In eq 6, *IC*_*MM*_ is the incremental cost of purchasing a MM over a boosted-SI vehicle.
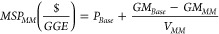
5
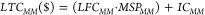
6
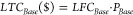
7
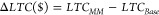
8

Lifetime emission differences were calculated by adding differences among cradle-to-refinery gate (*GHG*_*R*_), distribution (*GHG*_*D*_), and vehicle combustion (*GHG*_*C*_) emissions. Refinery gate emissions (*GHG*_*R*_) are those associated with feedstock growth, collection, transportation, and processing within the refinery which include all emissions produced up until products leave the refinery and are calculated by the Refinery Products Life Cycle Assessment (RP-LCA) model as discussed in Section 2.3. The RP-LCA model was designed to quantify the emissions impacts of integrating biofeedstocks into petroleum refineries, so negative credits for biomass production are included in the refinery gate emissions. The difference in refinery gate emissions over the vehicle lifetime (Δ*LTE*_*R*_) was calculated using eq 9 where *GGE*_*MM*_ is the quantity of MM gasoline-gallon equivalents produced each day by the refinery.
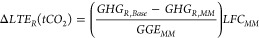
9

The distribution emission difference (Δ*LTE*_*D*_) once leaving the refinery gate was calculated with eq 10 with a constant emissions factor () pulled from The Greenhouse Gases, Regulated Emissions, and Energy Use in Transportation Model (GREET) associated with conventional gasoline.^11^
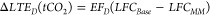
10

Combustion emission factors (*EF*_*C*_), tabularized in Table S1, were sourced for each MM bioblendstock (BBS) and fossil gasoline before oxygenate blending (BOB).^9^ Eqs 11^11^ and 12 were then used to determine blended emission factors for the base boosted-SI (10 vol % ethanol) and MM gasolines.
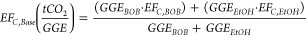
11
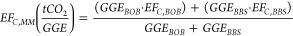
12

As a result, the lifetime difference in combustion emissions was calculated by using eq 13.
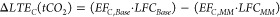
13

Finally, the total lifetime emission difference and the corresponding MAC were determined using eqs 14 and 15.
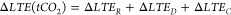
14
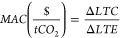
15

Models, tools, and data from prior Co-Optima research were combined to extract values for each equation to ultimately calculate MAC. Descriptions of each value source are given below.

### 2.2 Multi-Mode Gasoline Properties

Experimentally determined research octane numbers (*RON*_*Blend*_) and sensitivities (*S*_*Blend*_) were collected from the Co-Optima Fuel Properties Database for use in eq 1 to predict MM vehicle efficiency gains.^12^ These data were also used to create effective blending models consistent with a prior methodology^4^ to accurately reflect the nonlinear blending characteristics of MM bioblendstocks, due to their polarity, in the refinery model. Nonlinear blending models were developed for RON, Sensitivity, Reid Vapor Pressure (RVP), and ASTM D86% recovered distillation temperatures (T10, T50, and T90) using the data in Table S2.

### 2.3 Refinery Modeling Framework

A collection of refinery nonlinear programming (NLP) models were developed within AspenTech’s Process Industry Modeling System (PIMS) software package in a manner consistent with refining industry standards to optimize crude purchases and operations.^13^ The “Gulf-Coast” example model was customized to (1) represent a typical high-conversion, U.S. refinery located in Petroleum Administration for Defense District 3 (PADD3) region, (2) constrain finished products after blending to all pertinent ASTM specifications, and (3) represent all MM bioblendstocks with experimentally determined properties.^12^

**Figure 1:**
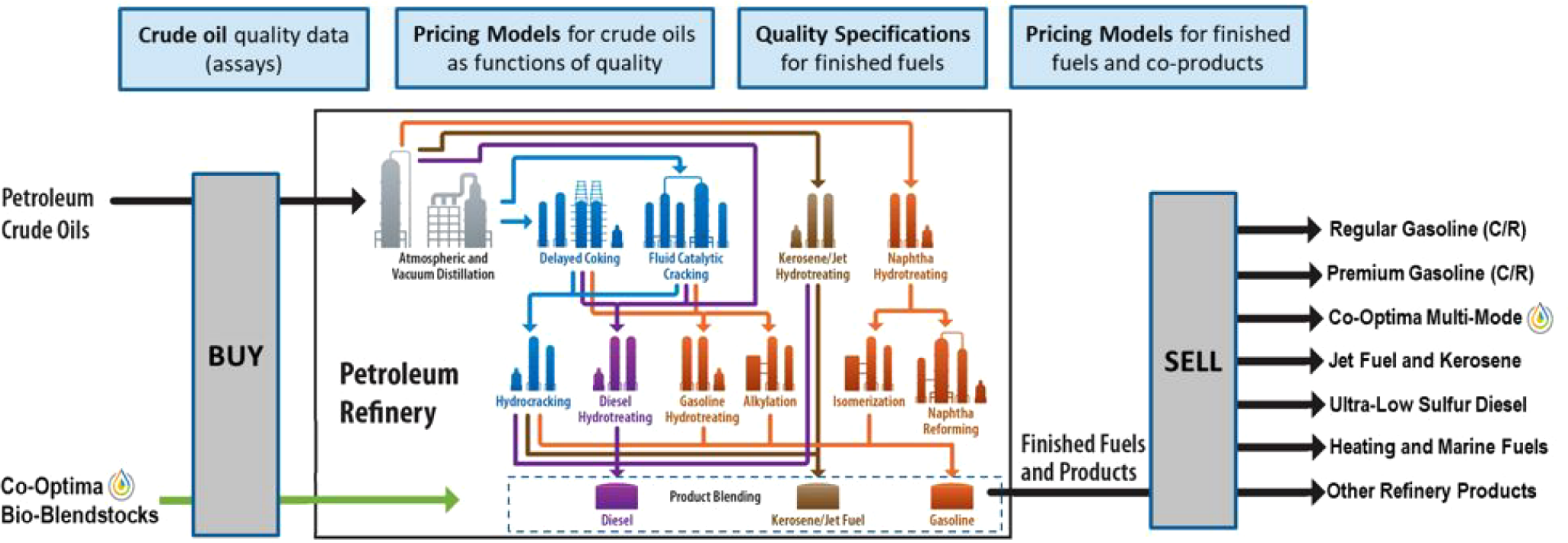
A graphical depiction of the refinery modeling framework composed of a central petroleum refinery optimization model built in Aspen-PIMS being fed realistic inputs including crude oil assays, input/output pricing, and fuel specifications from peripheral models.

Additionally, peripheral models were developed based on industrial data sources to feed the PIMS model realistic information. Feedstock/product prices, including *P*_*base*_, were modeled as functions of a benchmark West Texas Intermediate (WTI) crude price. Product demands were modeled by year, extending to 2050, based on scaled predictions given in the Energy Information Administration’s (EIA) Annual Energy Outlook (AEO) or the National Renewable Energy Laboratory’s (NREL) Automotive Deployment Options Projection Tool for gasoline (ADOPT).^10,14^ Aforementioned nonlinear blending property models were also constructed to determine effective blending properties as a function of the MM bioblendstock blend level. The PIMS and peripheral models were combined to form the refinery modeling framework, graphically depicted in Figure 1, used in this analysis and others where more detailed descriptions can be found.^4,15^

One alteration made to the modeling framework for this analysis was the inclusion of minimum selling prices (MSP) for each MM bioblendstock, tabularized in Table S1, determined through rigorous process simulation and techno-economic analysis (TEA) with n^th^ plant assumptions in prior Co-Optima research.^9^ Instead of calculating the maximum price a refiner would pay for each bioblendstock (break-even value) as in other works,^4,15^ TEA informed estimates for bioblendstock prices were implemented to determine *GM*_*Base*_ and *GM*_*MM*_ to calculate the minimum price at which refiner would need to sell MM gasoline (*MSP*_*MM*_) using eq 5. The refinery model also produces mass balances from which product energy flows (*GGE*_*MM*_, *GGE*_*BOB*_, *GGE*_*EtOH*_, and *GGE*_*BBS*_) were extracted.

### 2.4 Life-Cycle Analysis Model

**Figure 2:**
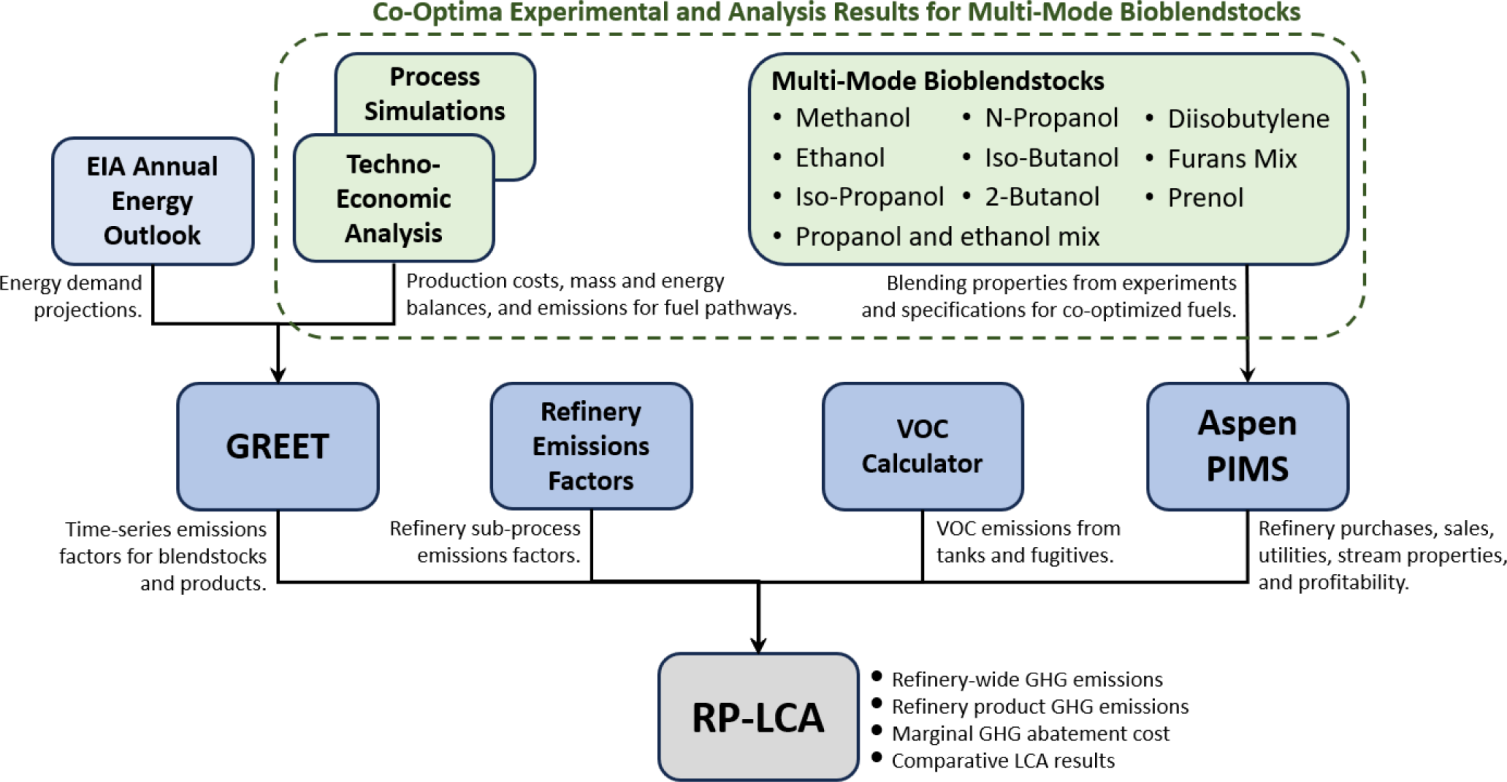
A high-level, graphical overview of the Refinery Products Life-Cycle Assessment Tool with data sources, flows, and outputs.

The Refinery Products Life Cycle Assessment (RP-LCA) model, developed by Argonne National Laboratory (ANL), was used to determine (*GHG*_*R*_), the environmental impacts from integrating Co-Optima MM bioblendstocks into refinery gasoline production and blending operations. The RP-LCA model is an Excel-based tool designed to link process-level material and energy flows from PIMS model solutions with appropriately sourced LCA data. Emission factors within RP-LCA are pulled from ANL’s Greenhouse Gases, Regulated Emissions, and Energy Use in Transportation Model (GREET) and an updated version of the Petroleum Refinery Life Cycle Inventory Model (PRELIM) for refinery subprocesses.^16,17^ RP-LCA results are given as 100-year global warming potentials as calculated in the International Panel on Climate Change’s Fourth Assessment Report in terms of tons of CO_2_-equivalent.^18^ The system boundary imposed in RP-LCA is a cradle-to-refinery gate, so emissions from the full supply chain of each refinery input are accounted for including feedstock collection, transportation, and processing. From the refinery-gate emissions calculated by RP-LCA (*GHG*_*R*_), distribution (*GHG*_*D*_) and vehicle combustion (*GHG*_*C*_) emissions were added to calculate cradle-to-grave emissions for each MM gasoline blend. Emission credits are included in RP-LCA for renewable and circular feedstocks to appropriately account for their decarbonization impacts at the refinery gate. Changes in utilities, such as heating, cooling, electricity, hydrogen, and wastewater treatment, are also captured. Moreover, market projections including U.S. electricity grid composition, pertinent technology improvements, and the same transportation fuel demand projections used in the PIMS model are included to give emission factors time sensitivity. Figure 2 shows a high level overview of the RP-LCA model and more details can be found in a forthcoming publication.^19^

### 2.5 Monte Carlo Input Variables

**Figure 3:**
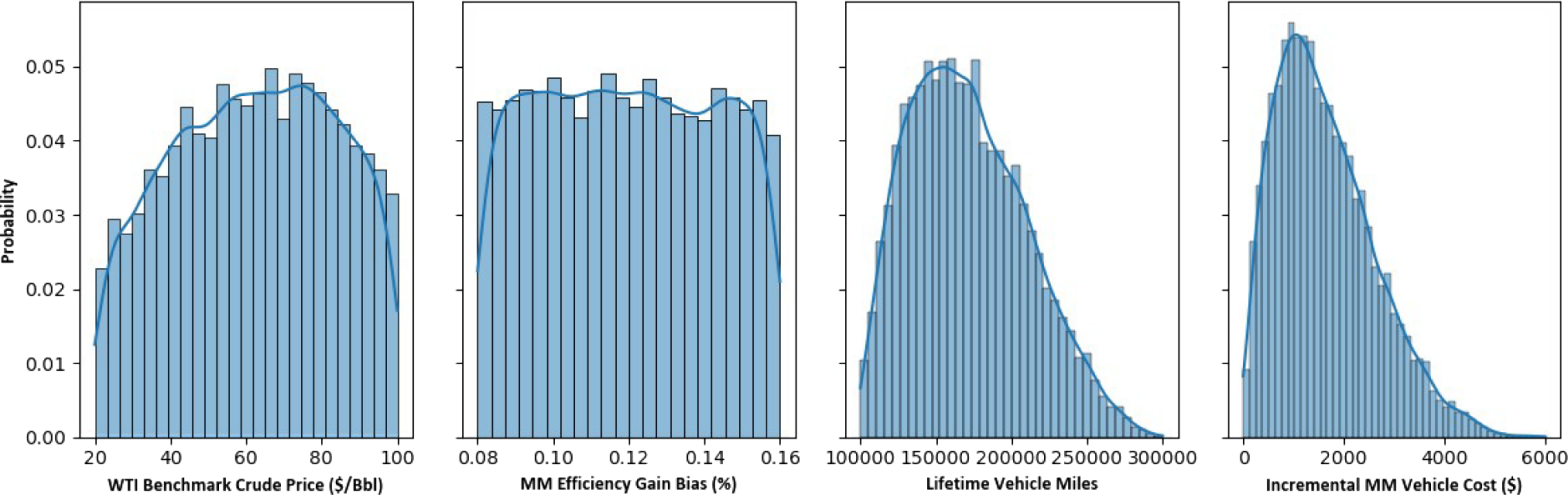
Distributions of the random variables considered during Monte Carlo simulation.

Key input variables used to calculate MAC included MM bioblendstock blend-levels, years, and WTI benchmark prices for the refinery model and the efficiency bias term (*EEF*_*Bias*_), lifetime vehicle miles (*LVMT*), and incremental MM vehicle cost (*IC*_*MM*_). Blend-levels and years were specially selected as case variables of interest. Consequently, refinery integration dynamics and LCA implications could be observed across varying degrees of decarbonization, set by blend-level, and varying market demands referenced from the EIA’s AEO, which are indexed by year as mentioned in Section 2.3. The other inputs carried uncertainty and, therefore, were treated as random variables within the context of Monte Carlo simulation. Handling uncertainties with Monte Carlo simulation was desirable for several reasons. First, although uncertain, each input variable’s range and distribution were well understood through prior Co-Optima research or historical data so reasonable estimations of distributions could be made. Second, Monte Carlo simulation produced a valuable coproduct in the form of MAC distributions that provided a risk profile associated with each MM bioblendstock candidate. Finally, simulating many randomized scenarios generated a large enough data set to assess variable importance’s which provided a better understanding of what inputs could be most influential in the successful deployment of MM technologies. The distributions shown in Figure 3 were determined on a case-by-case basis given the intricacies of each underlying variable as discussed below.

WTI year-overyear returns were assumed to follow a normal distribution, implying prices follow a log-normal distribution as traditionally assumed in most financial literature.^20^ Using historical WTI prices (*P*_*WTI*_) spanning 1990 to 2020 in yearly intervals from the EIA,^21^ the normal distribution (*N*) of returns was found to have a mean (μ) of 1.06 and standard deviation (σ) of 0.24. Using these parameters, yearly prices were projected from a starting value of $ 60/Bbl, as shown in eq 16. Bounds of [20, 100] ($/Bbl) were imposed to stay within the feedstock/product pricing model range, which is also why an artificial, bisecting starting price of 60 ($/Bbl) was chosen.


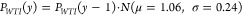
16

If  or , resample

For all  with 

Co-Optima’s High-Performance Fuels (HPF) Team estimated the merit function’s bias term (*EFF*_*Bias*_) to be somewhere between 8% and 16%, with high uncertainty.^8^ Therefore, *EFF*_*Bias*_ values were randomly sampled from a uniform distribution with bounds [0.08, and 0.16]. For lifetime vehicle miles, a modal value of 150,000 and upper bound of 300,000 miles for an exceptionally well maintained vehicle were pulled from literature, while a reasonable lower bound of 100,000 miles was imposed.^22,23^ A beta distribution (*α* = 2, *β* = 4) was fit to these values to fill in the asymmetric distribution. Finally, unpublished Co-Optima research has estimated incremental MM vehicle cost to range between $0 and $3,000 relative to comparable boosted spark ignition vehicles. However, a more conservative upper bound of $6,000 was implemented, while the median estimate of $1,500 was preserved as a modal value. Another beta distribution (*α* = 2, *β* = 5) was fit to these estimates to model incremental MM vehicle costs.

### 2.6 Variable Importance Determination

It was desirable to better understand which inputs and bioblendstock characteristics were most impactful in determining MACs. A byproduct of training some machine learning models is information regarding relative predictor variable importance. In particular, the average improvement in performance measure added by each variable split point after training a decision tree is a well-documented method to rank variable importance.^24^ Therefore, a gradient-boosted decision tree was trained to predict MAC based on the dependent variables presented in eqs 1–15 with the popular Python package XGBoost.^25^ To train the model, 90,000 Monte Carlo samples were used as data points, predictors were centered and scaled, and 20% of the data set was reserved for testing. The XGBoost regression model was trained using 5-fold cross validation to avoid overfitting. The R-squared score of the trained model in the testing set was 0.76. Finally, the average improvements in mean-square error (MSE) provided by splits along each variable within the decision tree were averaged and ranked.

## 3 Results

### 3.1 Marginal Abatement Cost Distributions

**Figure 4:**
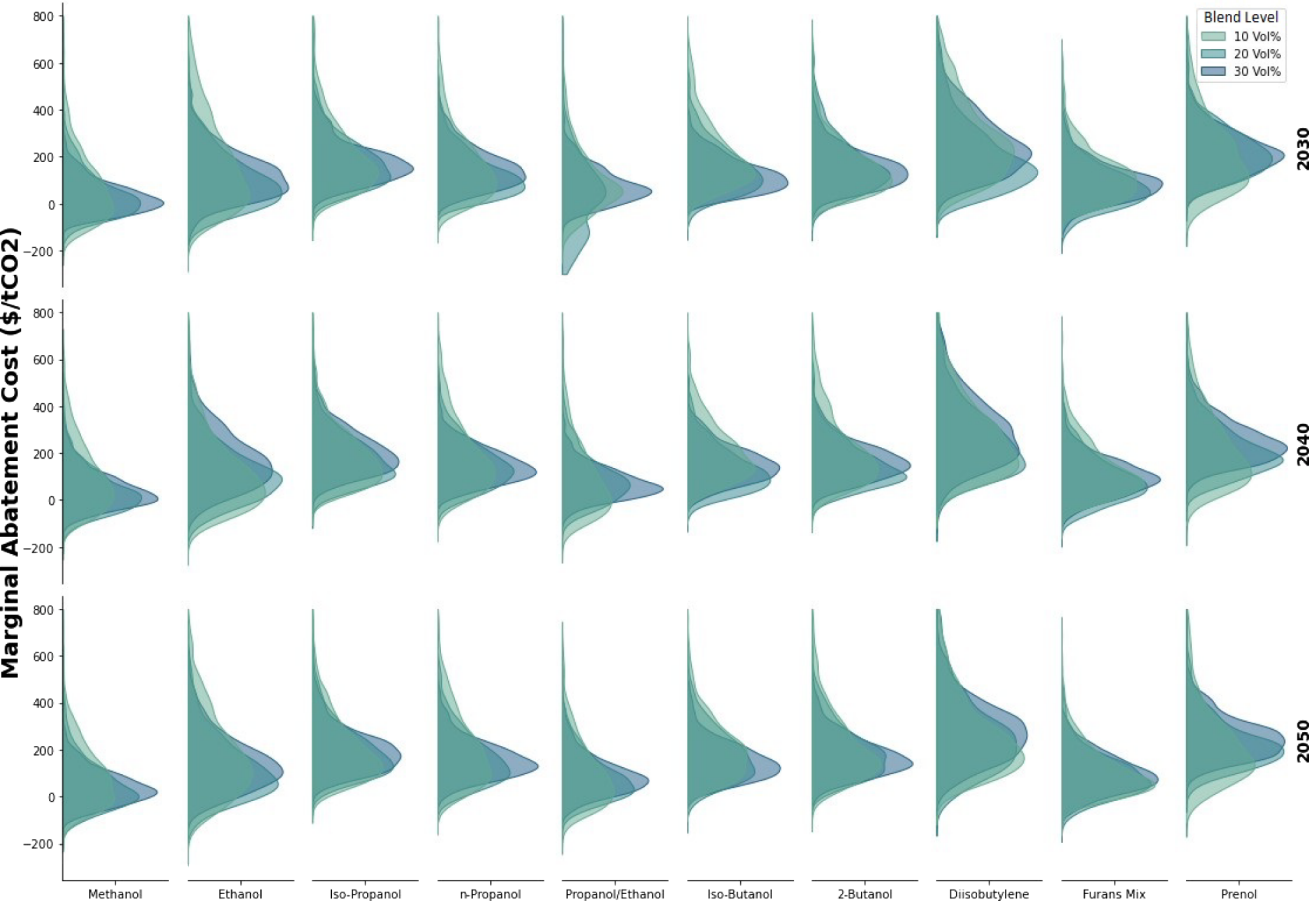
Distributions of marginal abatement costs (MACs) of CO_2_ per U.S. ton corresponding to different blend levels (blnlvl) of 10, 20, and 30 vol % across MM bioblendstocks and years (2030, 2040, and 2050), which correspond to different refinery product demand projections.

MACs for each MM bioblendstock candidate were calculated as shown in eqs 1–15 by fixing both blend level and year and then randomly sampling WTI prices, efficiency bias term (*EFF*_*Bias*_), lifetime vehicle miles (*LVMT*), and incremental MM vehicle cost (*IC*_*MM*_) values from the distributions depicted in Figure 3. Figure 4 shows MAC distributions resulting from 1,000 Monte Carlo samples for each MM bioblendstock at 10, 20, and 30 vol % blend levels for years 2030, 2040, and 2050 where random variables were resampled for each MAC distribution to mitigate sampling biases and distributions were smoothed using kernel density estimation (KDE) with a bandwidth parameter of 1.0.^26^ MAC value means and standard deviations are tabulated in a heatmap in Table S3 for reference.

Methanol, ethanol, and propanol/ethanol stood out as having consistently low MACs with averages of 61, 142, and 87 $/tCO_2_ across blend levels/years while di-isobutylene, prenol, and iso-propanol had the highest with averages of 265, 224, 194 $/tCO_2_. For most bioblendstocks, the 10 vol % blending level produced the most variability which seemed to consistently shrink with increasing blend level. Additionally, higher blend levels appeared to shift each MAC distribution backward, indicating that generally, the costs of MM gasoline production increased more quickly than their decarbonization impact when scaling from 10 to 30 vol %, though only slightly. This trend follows results from a similar analysis regarding boosted-SI Co-Optima gasolines which found that bringing in additional volumes of high RON and sensitivity blendstocks had marginal benefits to refineries beyond the 10 vol % blend level.

Visualizing the data in Figure 4, and in the alternative heatmap format provided in Table S3, shows some inconsistencies in trends observed across blend-levels and years. This is a phenomenon also observed in similar refinery optimization analysis that can be attributed to the fact the model is optimizing purely based on costs and CO_2_ abatement is calculated afterward.^27^ Consequently, the optimizer has a tendency to shift its crude slate when adapting to changing bioblending levels or product demands which can cause step-function changes in refinery emissions (*GHG*_*R*_), which ultimately impact calculated MACs. Considering LCA metrics more directly in the objective function would result in smoother transitions across average crude slate carbon intensities when considering different blend levels and years/product demands. Not accounting for LCA metrics in the optimizer’s objective function could be considered a limitation, but in practice refineries make decisions to optimize overall profitability and, in the absence of policy incentives to produce MM gasolines with low carbon intensities, optimizing overall gross margin could also be considered a more realistic scenario.

### 3.2 Variable Importance

**Figure 5:**
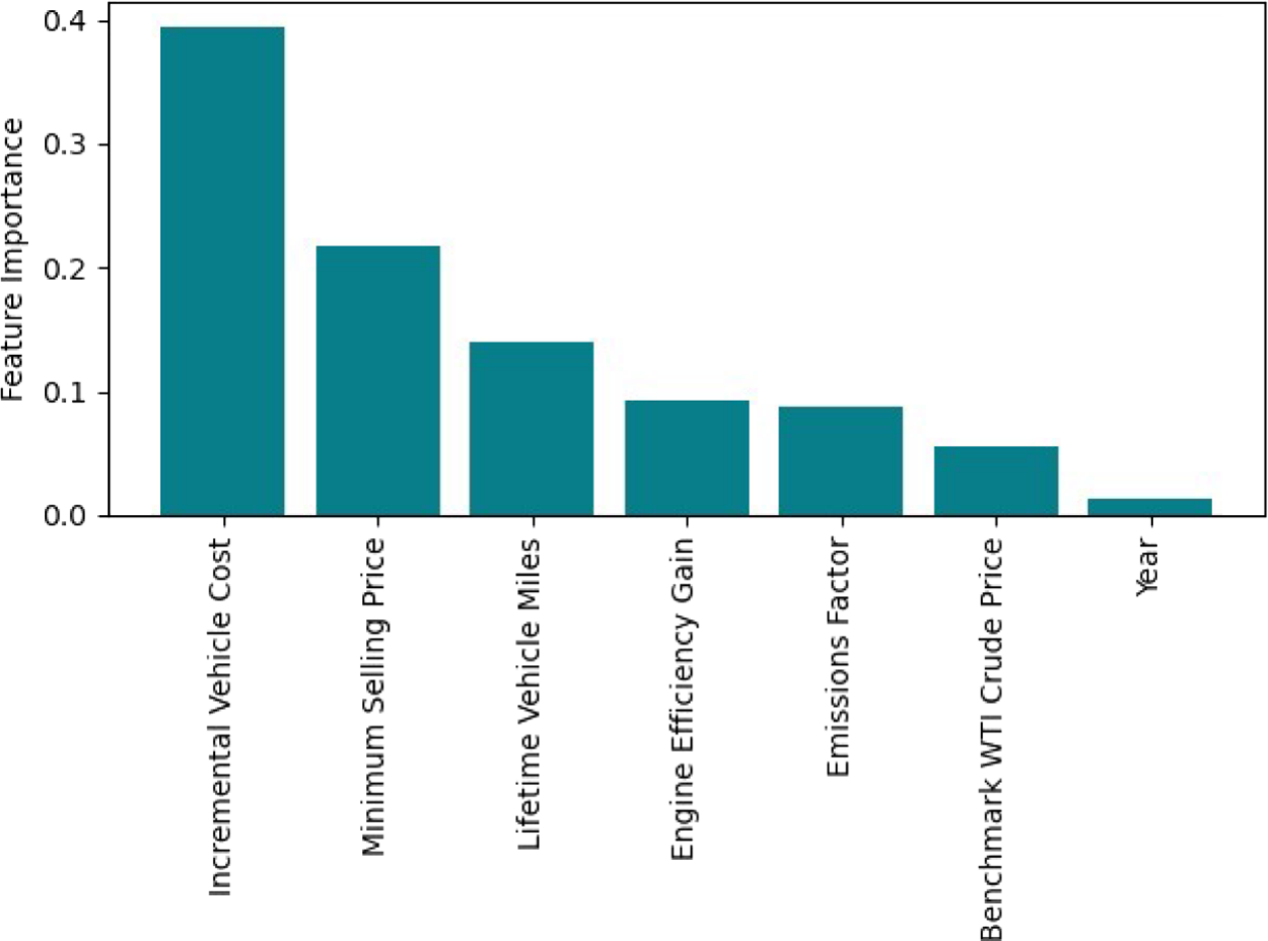
Relative importance of each variable (feature) that influences the MAC associated with Co-Optima MM fuels and engines. Variables in ranked order of highest to lowest importance included (1) incremental cost of the MM vehicle, (2) minimum selling price of the MM bioblendstock, (3) lifetime miles covered by the hypothetical vehicle, (4) efficiency gain provided by the MM fuel and engine, (5) emission factor of the MM bioblendstock, (6) the benchmark WTI crude oil price, and (7) year of calculation [2025, 2030, 2035, 2040, 2045, 2050] setting refinery product demands.

Figure 5 shows the relative variable importance, calculated as the unitless average of how well splitting on each variable improved the decision tree prediction’s mean-square error (MSE), of each variable influencing MAC. Although bioblendstock minimum selling price was not explicitly used in eqs 1–15, it was an input to the PIMS and RP-LCA models.

Figure 5 indicates that price reductions, in either incremental vehicle costs or MM bioblendstock production costs, would have the most impact in reducing MACs. This assertion is also supported by methanol, ethanol, and propanol/ethanol producing the most favorable MAC distributions in Figure 4 while having the lowest MSPs. Moreover, increasing lifetime vehicle miles was found to be the next most important modification that would increase the overall emissions savings provided by an MM vehicle. Also, increasing efficiency gains, as calculated in eq 1, and reducing emissions factors would have more moderate impacts on reducing the MAC.

### 3.3 Marginal Abatement Cost Comparisons

**Figure 6:**
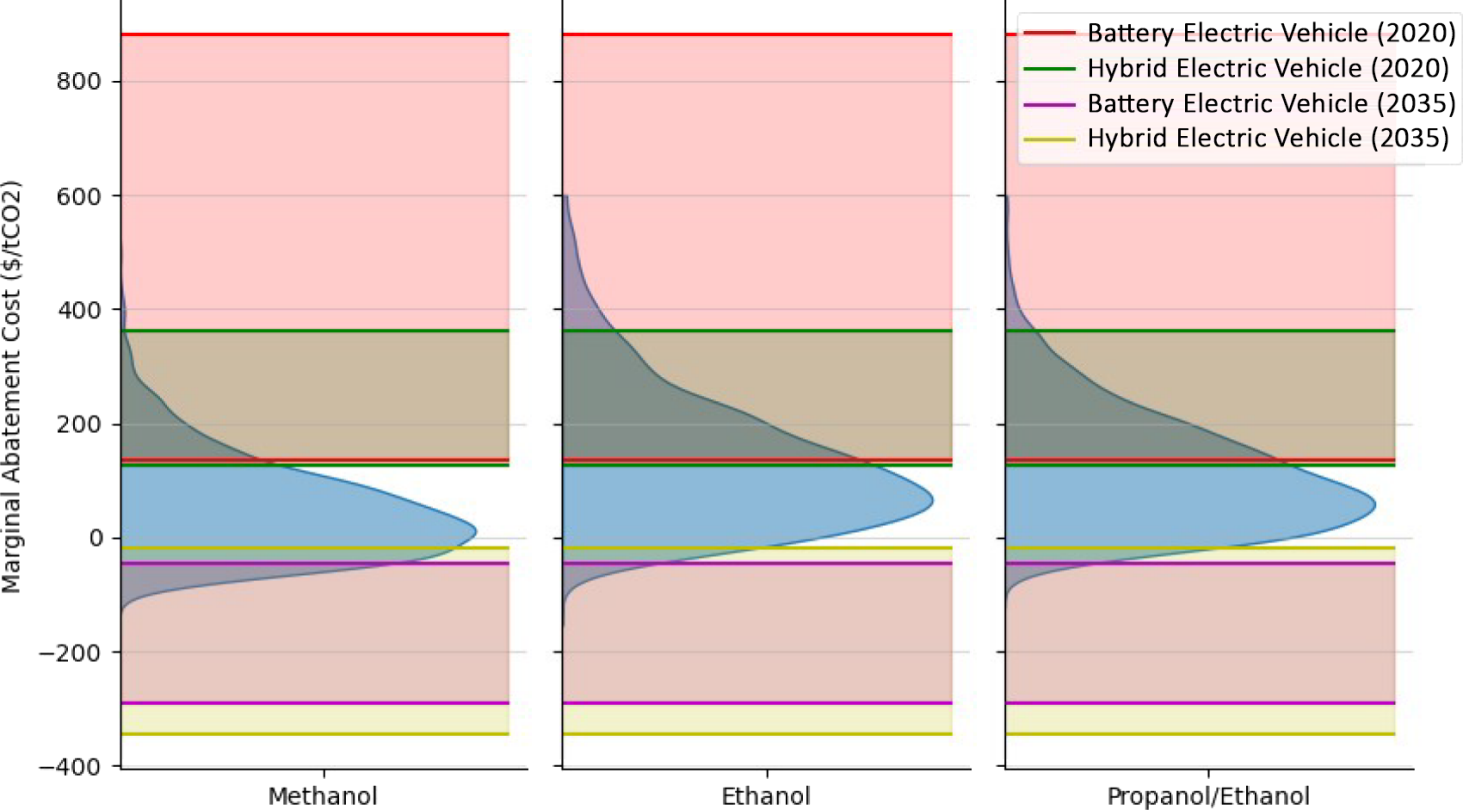
Marginal abatement cost (MAC) of CO_2_ ($/U.S. Ton) distributions for methanol, ethanol, and propanol/ethanol mixture blended at 20 vol % in year 2030 displayed alongside MAC ranges for battery electric vehicles (BEV) and hybrid electric vehicles (HEV) given current (2020) and future (2030) average U.S. grid mix assumptions, all calculated using consistent LCA data and methods from the GREET model.^17,28^.

To give context to the MACs presented in Figure 4, comparisons with alternative light-duty vehicle decarbonization technologies are shown in Figure 6. MAC simulations were generated for the three MM gasoline blends with the lowest MACs from Figure 4 (methanol, ethanol, and propanol/ethanol) all at 20 vol % in year 2030 with 2500 samples a piece. Comparisons were made with battery electric vehicles (BEV) and hybrid electric vehicles (HEV) MAC range estimates previously calculated and reported using the GREET model to maintain consistency between underlying LCA methods and assumptions.^28^ Moreover, individual MAC ranges encompass 200-to-400-mile battery ranges for BEVs and HEV to plug-in HEV (PHEV) vehicles, which are midsize sedans to match the underlying base efficiency assumption used in the MM gasoline MAC calculations. Distinct MAC ranges for BEVs/HEVs are provided using current (2020) and future (2035) average U.S. grid mix LCA assumptions to analyze how MM gasoline might compare to electrified vehicles over time as the grid decarbonizes.

Figure 6 suggests that select MM gasoline blends would likely be a more cost competitive light-duty vehicle decarbonization strategy than electrification based on the current (2020) average U.S. grid mix carbon intensity. However, as the grid decarbonizes, BEVs/HEVs will be more likely to provide low or even negative MACs. Therefore, the results suggest that MM gasolines would best serve the market as an interim strategy to partially decarbonize the light-duty transportation fleet, as the investment and capital projects needed to decarbonize the grid and improve HEV/BEV MACs occur over time.

## 4 Discussion

Results from Figures 4 and 6 indicate that some Co-Optima MM gasoline blends have the potential to provide cost-competitive GHG emission reductions, particularly, while the grid and BEVs/HEVs decarbonize over time. However, simulated distributions also show appreciable risks of higher lifetime MACs which could be best mitigated through incremental MM vehicle cost and bioblendstock production cost reductions as suggested by Figure 5. Moreover, Co-Optima technology readiness, preexisting gasoline distribution infrastructure, and refinery interest in decarbonization opportunities could allow MM GHG reductions to scale more quickly when compared with alternative light-duty vehicle decarbonization technologies. More specifically, Figure 5 indicates Co-Optima bioblendstock prices and emission factors are influential variables in determining lifetime MACs, so more mature, and consequently lower cost MM gasoline blends with methanol, ethanol, and propanol/ethanol mixtures are particularly well-poised to provide low-cost emissions reductions within a short time span.

Neat methanol has been used successfully in racing vehicles, with minimal range requirements thereby mitigating the fuel’s lower energy density, because of its high octane and fast flame velocity.^29^ However, methanol’s low-energy density and high hygroscopicity (ability to attract and absorb water), making large-scale distribution and storage more difficult, have prevented its wider-spread adoption as a gasoline blendstock as evidenced by E.U. and U.S. maximum blending limits of 3 and 0.3 vol %, respectively.^30,31^ Nevertheless, large-scale methanol gasoline blending has been demonstrated in countries like China, producing between 0.9 and 1.5 billion gallons of methanol per year with national guidance to invest more into methanol production, infrastructure, and vehicle technologies.^30,32^ Methanol is also a favorable platform chemical for several biomass conversion technologies, and marine fuels, which could provide additional incentive to invest in its distribution infrastructure.^33^

Additionally, the ethanol-based MM blendstocks (ethanol and propanol/ethanol mixture) are advantaged by the fact 10 vol % ethanol blending is already standardized in the U.S. gasoline market with higher blends (E15, E85, etc.) also distributed widely. Therefore, supply, distribution, and infrastructure investment constraints would likely be less restrictive for ethanol-based MM gasolines when compared to other blendstocks with U.S. production capacity estimated to be 17.7 billion gallons of ethanol per year.^34^ Moreover, the federal incentives, such as the U.S. Department of Agriculture’s (USDA) Higher Blends Infrastructure Incentive Program (HBIIP), already provide financial assistance for infrastructure projects aiming to increase ethanol, or other biofuel, blending levels.^34^ Supply constraints would be further alleviated if BEV market adoption progresses as anticipated, leading to a 17% (AEO) or 38% (ADOPT) drop in gasoline/ethanol demand.^10,14^ Ethanol producers could maintain sale volumes despite market headwinds by diversifying into markets such as sustainable aviation fuels (SAF) or 20, 30, or higher vol % MM gasoline blends.

While the other MM bioblendstocks yield less competitive, higher lifetime MACs, the uncertainty associated with their n^th^-plant scaled production costs are also much higher than the more matured methanol, ethanol, and propanol/ethanol processes. Demand stemming from MM gasoline blending could incentivize bioblendstock production and competition. Increasing cumulative production volumes have been linked to reductions in production costs through industrial learnings as exemplified by the nearly 70% reduction in ethanol prices in Brazil from 1980 to 2002.^35^ Learning curve mechanisms could reduce bioblendstock production costs in unanticipated ways and allow higher-quality blendstocks, in terms of RON and sensitivity, such as furans or prenol, to become economically feasible. Additionally, hard-to-anticipate technological improvements in the biomass conversion pathways underpinning each MM bioblendstock could improve their associated MACs with continued interest and R&D.

MM gasoline deployment would face several risks that could influence each bioblendstock’s success in the market. First, production volumes would need to be sufficiently large to reduce bioblendstock costs to their n^th^-plant estimates, a highly influential factor in determining lifetime MACs. Second, Figure 5 identified the incremental vehicle cost of MM engines over standard boosted-SI engines as the most influential factor in predicting MACs. Vehicle manufacturers would need to limit MM vehicle R&D and manufacturing costs for the technology’s deployment to result in cost-competitive MACs. Third, this study is limited in capturing the infrastructure investment costs that would be needed to distribute each bioblendstock, though they should be lower than alternatives like HEVs/BEVs since sufficient liquid distribution infrastructure already exists throughout the U.S. Since costs, whether they be associated with bioblendstock or vehicle production, were found to be more influential than other variables, unforeseen infrastructure costs such as metallurgy or seal material changes would likely be equally influential and could pose risks to the technology’s deployment.

Finally, the MM fuels presented herein are limited in being unable to achieve net-zero emissions because of their reliance on blending with petroleum fuels. In contrast, HEV and BEV adoption is predicated upon the continual decarbonization of the U.S. power grid overtime until net-zero transportation is eventually achieved.^1^ Further MM bioblendstock conversion technology developments including higher conversion efficiencies, carbon sequestration technology implementations, and higher blending volumes up to neat fuels would need to occur to reach net-zero, or possibly net-negative emission status. However, Figure 6 suggests that commercializing MM gasolines could be a cost-competitive option to decarbonize the light-duty fleet as the grid decarbonizes over time, and electrified vehicles can provide net-zero transportation options at scale.

## 5 Conclusion

In summary, optimizable refinery models were developed to quantify the costs and benefits of integrating renewable bioblendstocks with existing refining infrastructure and producing high-quality fuel blends for advanced, high-efficiency MM engines. The results of the analysis, based largely on experimental fuels research data from Co-Optima, show that bioblendstock integration can contribute to attractive decarbonization strategies. Combining the efficiency gains from advanced fuel engines with the reoptimization of refinery operations for producing Co-Optima fuel blends results in probabilistic averages for marginal CO_2_ abatement costs nearing zero for several bioblendstocks with blend levels up to 30%. Moreover, existing infrastructure could reduce technology deployment timelines, specifically for mature biofuels such as methanol, ethanol, or a propanol/ethanol mixture. However, risks associated with bioblendstock production and incremental MM vehicle costs coupled with the technology’s inability to reach net-zero emissions when limited to lower blending volumes could deter the investment needed for market development.

Given the opportunities and obstacles associated with Co-Optima MM technology deployment, MM vehicles and gasolines could have a role to play in providing valuable, near-term GHG emission reductions as the U.S. transitions away from fossil fuels. Although there are many light-duty vehicle decarbonization strategies competing for investment, such as e-fuels, alternative biofuels, or HEV/BEVs, few appear as readily scalable. For example, in regions of the U.S. with sufficient E15 gasoline supply, MM gasoline production and distribution infrastructure already exists, and future MM vehicles sold and operated in those regions could immediately capitalize on increased engine efficiencies. Furthermore, MM gasolines could reduce emissions in sectors that are challenged or resistant to electrification, such as performance, powersport, or long-range vehicle applications.

## Supporting Information Available

Multi-Mode bioblendstock data used as the basis for analysis and tabularized version of calculated marginal abatement costs of CO_2_ (PDF)

## Abbreviations

ADOPT: Automotive Deployment Options Projection Tool
AEO: Annual Energy Outlook
ANL: Argonne National Laboratory
ASTM: American Society for Testing and Materials
BOB: Before Oxygenate Blending
BEV: Battery Electric Vehicle
DAC: Direct Air Capture
EIA: Energy Information Administration
GHG: Greenhouse Gas
GREET: Greenhouse Gases, Regulated Emissions, and Energy Use in Transportation
HBIIP: Higher Blends Infrastructure Incentive Program
HPF: High Performance Fuels
IEA: International Energy Agency
INL: Idaho National Laboratory
KDE: Kernel Density Estimation
LCA: Life Cycle Assessment
MAC: Marginal Abatement Cost
MM: Multi-Mode Ignition
MON: Motor Octane Number
MPG: Miles Per Gallon
MSE: Mean Squared Error
MSP: Minimum Selling Price
NLP: Nonlinear Programming
NREL: National Renewable Energy Laboratory
PADD: Petroleum Administration Defense District
PIMS: Process Industry Modeling System
PNNL: Pacific Northwest National Laboratory
PRELIM,: Petroleum Refinery Life Cycle Inventory Model
RON: Research Octane Number
RP-LCA: Refinery Products Life Cycle Assessment
RVP: Reid Vapor Pressure
SAF: Sustainable Aviation Fuel
SI: Spark Ignition
TEA: Techno-economic Analysis
USDA: United States Department of Agriculture
WTI: West-Texas Intermediate
