# Supplementary material for: A Comprehensive Assessment of the Marginal Abatement Costs of CO2 of Co-Optima Multi-Mode Vehicles
Source: Energy Fuels. 2024 Dec 19;39(1):444–53. doi: 10.1021/acs.energyfuels.4c03451 (PMC11726429; doi:10.1021/acs.energyfuels.4c03451)
Supplement: Supplementary file 1 — ef4c03451_si_001.pdf [file ef4c03451_si_001.pdf]

# A Comprehensive Assessment of the Marginal Abatement Costs of CO2 of Co-Optima Multi-Mode Vehicles

*Nicholas A. Carlson*[1], Michael S. Talmadge[1], George G. Zaimes[2], Troy R. Hawkins[2], Yuan Jiang[3]*

[1]National Renewable Energy Laboratory, 15013 Denver West Parkway, Golden, CO 80401

[2]Argonne National Laboratory, 9700 Cass Avenue, Lemont, IL 60439

[3]Pacific Northwest National Laboratory, 902 Battelle Boulevard, Richland, WA 99354

*nicholas.carlson@nrel.gov

***Table S1.*** Prices, energy densities, and combustion emission factors for Co-Optima MM bio-blendstocks used to calculate marginal abatement costs of $CO_2$.[1,2]

| Blendstock | Minimum Selling Price ($/Gal) | Lower Heating Value (MJ/L) | Life Cycle GHG Emissions ($gCO_2$/MJ) | Combustion Emission Factor ($gCO_2$/MJ) |
|---|---|---|---|---|
| **Methanol** | 1.21 | 14.3 | 9.9 | 75.4 |
| **Ethanol** | 2.53 | 21.3 | 11 | 71.0 |
| **Iso-Propanol** | 3.29 | 21.7 | 27 | 79.6 |
| **N-Propanol** | 3.19 | 22.5 | 31 | 78.8 |
| **Propanol and Ethanol Mixture** | 2.02 | 19.7 | 15 | 78.8 |
| **Iso-Butanol** | 3.15 | 24.0 | 21 | 79.5 |
| **2-Butanol** | 3.37 | 24.1 | 31 | 79.5 |
| **Diisobutylene** | 4.19 | 28.7 | 72 | 78.2 |
| **Furans Mixture** | 3.64 | 31.6 | 33 | 73.9 |
| **Prenol** | 4.85 | 27.0 | 34 | 82.2 |
| **Fossil BOB** | | 28.4 | 90 | 80.0 |

***Table S2.*** Blending properties of Co-Optima MM bio-blendstocks from Fuels Properties Database.[3] Note that multiple datapoints pertaining to different fossil-blends (before-oxygenate blends (BOB)) are given for each bio-blendstock at each blend-level. The effective blending properties are averaged over the different BOB blends.

| Bio-Blendstock | Blend Vol.% | Blending MON | Blending RON | Blending Sensitivity | Blending RVP (kPa) | Blending T10 (°F) | Blending T50 (°F) | Blending T90 (°F) |
|---|---|---|---|---|---|---|---|---|
| **Methanol** | 10% | 88.5 | 96.4 | 7.9 | 60.9 | 51.4 | 101.3 | 167.2 |
| | 20% | 89.5 | 100.9 | 11.4 | 60.2 | 58.7 | 65.9 | 172.2 |
| | 30% | 90.4 | 102.6 | 12.2 | 60.2 | 52.7 | 60.9 | 158.4 |
| | Neat BBS | 89 | 109 | 20 | 12.3 | - | - | - |
| **Ethanol** | 10% | 86.5 | 94.0 | 7.5 | 67.2 | 50.9 | 83.8 | 157.5 |
| | 20% | 88.6 | 99.1 | 10.4 | 65.5 | 55.8 | 74.2 | 158.2 |
| | 30% | 88.6 | 100.4 | 11.8 | 63.7 | 54.4 | 73.0 | 151.6 |
| | Neat BBS | 90.0 | 109.0 | 19.0 | 18.6 | - | - | - |
| **Iso-Propanol** | 10% | 85.8 | 92.0 | 6.2 | 62.8 | 54.0 | 86.5 | 157.3 |
| | 20% | 88.4 | 96.0 | 7.6 | 60.9 | 55.6 | 76.0 | 153.7 |
| | 30% | 91.0 | 99.3 | 8.3 | 58.6 | 57.2 | 77.2 | 151.2 |
| | Neat BBS | 96.7 | 112.5 | 15.8 | 14.6 | - | - | - |
| **N-Propanol** | 10% | 85.0 | 92.4 | 7.3 | 60.7 | 55.3 | 86.1 | 157.1 |
| | 20% | 86.4 | 95.9 | 9.5 | 58.7 | 57.3 | 86.0 | 154.4 |
| | 30% | 87.3 | 98.9 | 11.6 | 56.1 | 60.7 | 89.3 | 151.2 |
| | Neat BBS | 89.0 | 104.0 | 15.0 | 9.7 | - | - | - |
| **Propanol and Ethanol Mixture** | 10% | 82.8 | 92.0 | 9.2 | 39.4 | 63.1 | 100.2 | 168.3 |
| | 20% | 81.1 | 92.7 | 11.6 | 39.4 | 63.2 | 75.0 | 164.9 |
| | 30% | 81.1 | 94.3 | 13.2 | 38.4 | 64.2 | 75.6 | 164.9 |
| | Neat BBS | - | - | - | - | - | - | - |
| **Iso-Butanol** | 10% | 84.8 | 90.7 | 5.9 | 58.1 | 56.6 | 91.9 | 157.2 |
| | 20% | 86.4 | 94.6 | 8.2 | 55.4 | 62.8 | 98.0 | 157.5 |
| | 30% | 87.5 | 97.3 | 9.8 | 52.8 | 60.8 | 97.7 | 139 |
| | Neat BBS | 93.0 | 107.0 | 14.0 | 1.7 | - | - | - |

| | | | | | | | | |
|---|---|---|---|---|---|---|---|---|
| **2-Butanol** | 10% | 86.5 | 93.1 | 6.6 | 35.8 | 69.7 | 96.4 | 169.0 |
| | 20% | 88.2 | 96.1 | 7.9 | 34.1 | 77.8 | 98.4 | 170.4 |
| | 30% | 89.5 | 98.2 | 8.7 | 32.6 | 73.7 | 94.1 | 162.4 |
| | Neat BBS | 93.0 | 107.0 | 14.0 | 1.7 | - | - | - |
| **Diisobutylene** | 10% | 85.3 | 91.9 | 6.7 | 56.0 | 58.3 | 99.6 | 155.4 |
| | 20% | 86.2 | 95.6 | 9.4 | 51.4 | 65.2 | 104.0 | 155.2 |
| | 30% | 86.8 | 98.2 | 11.4 | 47.1 | 65.6 | 102.0 | 147.9 |
| | Neat BBS | 87.0 | 106.0 | 19.0 | 11.0 | - | - | - |
| **Furans Mixture** | 10% | 86.2 | 95.3 | 9.1 | 56.6 | 56.5 | 93.4 | 157.3 |
| | 20% | 86.7 | 99.2 | 12.5 | 53.9 | 61.5 | 93.8 | 157.1 |
| | 30% | 86.9 | 100.4 | 13.5 | 52.0 | 59.1 | 87.8 | 148.9 |
| | Neat BBS | 91.6 | 109.0 | 17.4 | 7.8 | - | - | - |
| **Prenol** | 10% | 84.9 | 92.4 | 7.5 | - | - | - | - |
| | 20% | 84.6 | 95.5 | 10.9 | - | - | - | - |
| | 30% | 84.1 | 96.8 | 12.6 | - | - | - | - |
| | Neat BBS | 74 | 93.5 | 19.5 | | - | - | - |

***Table S3.*** Marginal abatement cost of $CO_2$ distribution means and standard deviations for each Multi-Mode bio-blendstock blended at 10, 20, and 30 volume % blend levels with petroleum gasoline blendstocks in years 2030, 2040, and 2050. Distributions are composed of 1000 random samples of benchmark West-Texas Intermediate crude prices, Multi-Mode engine efficiency gain biases, lifetime vehicle miles, and incremental Multi-Mode vehicle costs drawn from the distributions depicted in **Figure 3**. Table entries are shaded such that lower means and standard deviations, which are preferable, are darker shades of green while higher values are darker shades of red.

| **Year** | **2030** | | | **2040** | | | **2050** | | |
|---|---|---|---|---|---|---|---|---|---|
| **Blend Level (Vol%)** | **10** | **20** | **30** | **10** | **20** | **30** | **10** | **20** | **30** |
| | | | | **Mean** | | | | | |
| **Methanol** | 77.24 | 52.54 | 37.33 | 86.42 | 27.80 | 52.54 | 92.07 | 54.28 | 64.77 |
| **Ethanol** | 154.13 | 108.45 | 114.89 | 127.27 | 142.72 | 162.69 | 169.55 | 118.06 | 175.96 |
| **Iso-Propanol** | 194.91 | 176.94 | 182.33 | 201.29 | 182.61 | 192.07 | 228.70 | 199.84 | 189.83 |
| **n-Propanol** | 161.56 | 142.53 | 152.68 | 167.10 | 175.83 | 171.29 | 196.00 | 172.25 | 171.27 |
| **Propanol/Ethanol** | 80.84 | 39.63 | 72.37 | 91.19 | 97.23 | 99.87 | 97.22 | 111.70 | 90.58 |
| **Iso-Butanol** | 175.94 | 170.35 | 120.44 | 214.51 | 147.80 | 147.23 | 204.91 | 160.07 | 148.65 |
| **2-Butanol** | 178.33 | 170.34 | 160.48 | 174.06 | 163.54 | 186.75 | 203.14 | 172.07 | 201.95 |
| **Diisobutylene** | 223.25 | 224.35 | 266.42 | 284.46 | 235.51 | 283.41 | 283.24 | 283.65 | 296.36 |
| **Furans Mix** | 108.54 | 78.82 | 99.66 | 148.64 | 96.80 | 109.51 | 117.60 | 82.43 | 109.91 |
| **Prenol** | 217.70 | 202.84 | 215.56 | 207.07 | 226.06 | 262.48 | 221.77 | 222.06 | 240.59 |
| | | | | **Standard Deviation** | | | | | |
| **Methanol** | 149.51 | 109.50 | 73.24 | 131.55 | 83.51 | 82.71 | 137.90 | 101.92 | 90.22 |
| **Ethanol** | 175.04 | 117.60 | 104.68 | 162.92 | 140.74 | 125.47 | 163.22 | 132.88 | 124.12 |
| **Iso-Propanol** | 140.96 | 104.19 | 91.26 | 135.94 | 108.87 | 88.29 | 144.56 | 114.75 | 91.56 |
| **n-Propanol** | 137.42 | 103.51 | 88.66 | 139.32 | 119.48 | 94.26 | 147.37 | 116.02 | 91.38 |
| **Propanol/Ethanol** | 133.53 | 169.18 | 74.39 | 146.30 | 105.33 | 82.62 | 140.32 | 106.48 | 80.96 |
| **Iso-Butanol** | 118.63 | 117.64 | 76.01 | 124.55 | 106.29 | 80.84 | 123.45 | 108.93 | 81.37 |
| **2-Butanol** | 131.80 | 124.86 | 84.30 | 122.26 | 113.26 | 89.96 | 132.25 | 105.15 | 101.34 |
| **Diisobutylene** | 142.95 | 151.49 | 136.55 | 164.35 | 142.14 | 132.49 | 162.44 | 164.68 | 138.68 |
| **Furans Mix** | 105.16 | 96.04 | 86.14 | 121.63 | 98.95 | 85.95 | 106.50 | 90.15 | 84.57 |
| **Prenol** | 154.22 | 100.00 | 90.15 | 151.00 | 108.24 | 95.55 | 154.94 | 107.58 | 79.27 |

## References

(1) Bartling, A. W.; Benavides, P. T.; Phillips, S. D.; Hawkins, T.; Singh, A.; Wiatrowski, M.; Tan, E. C. D.; Kinchin, C.; Ou, L.; Cai, H.; et al. Environmental, Economic, and Scalability Considerations of Selected Bio-Derived Blendstocks for Mixing-Controlled Compression Ignition Engines. *ACS Sustainable Chem. Eng.* **2022**, *10* (20), 6699–6712. https://doi.org/10.1021/acssuschemeng.2c00781.

(2) Benavides, P. T.; Bartling, A. W.; Phillips, S. D.; Hawkins, T. R.; Singh, A.; Zaimes, G. G.; Wiatrowski, M.; Harris, K.; Burli, P. H.; Hartley, D.; et al. Identification of Key Drivers of Cost and Environmental Impact for Biomass-Derived Fuel for Advanced Multimode Engines Based on Techno-Economic and Life Cycle Analysis. *ACS Sustainable Chem. Eng.* **2022**, *10* (32), 10465–10475. https://doi.org/10.1021/acssuschemeng.2c00944.

(3) *Co-Optimization of Fuels and Engines: Fuel Properties Database | NREL*. https://www.nrel.gov/transportation/fuels-properties-database/ (accessed 2020-12-07).
